# Supplementary material for: Nanophotonic neural probes for in vivo photostimulation, electrophysiology, and microfluidic delivery
Source: Microsyst Nanoeng. 2026 Mar 20;12:100. doi: 10.1038/s41378-026-01192-6 (PMC13002897; doi:10.1038/s41378-026-01192-6)
Supplement: Supplementary file 1 — Supplementary Information [file 41378_2026_1192_MOESM1_ESM.pdf]

Nanophotonic neural probes for *in vivo* photostimulation, electrophysiology, and microfluidic delivery

Xin Mu[1,2,3,*], Homeira Moradi Chameh[4], Mandana Movahed[4], Fu Der Chen[1,2,3], John N. Straguzzi[1], Piyush Kumar[1], Andrei Stalmashonak[1], Hannes Wahn[1], Hongyao Chua[5], Xianshu Luo[5], Guo-Qiang Lo[5], Joyce K. S. Poon[2,3], Taufik A. Valiante[2,3,4,6,7], and Wesley D. Sacher[1,3,*]

[1]Max Planck Institute of Microstructure Physics, Weinberg 2, 06120 Halle, Germany
[2]Department of Electrical and Computer Engineering, University of Toronto, 10 King's College Road, Toronto, Ontario M5S 3G4, Canada
[3]Max Planck-University of Toronto Centre for Neural Science and Technology, Toronto, Ontario, Canada
[4]Krembil Brain Institute, University Health Network, Toronto, Ontario, Canada
[5]Advanced Micro Foundry Pte. Ltd., 11 Science Park Road, Singapore Science Park II, 117685, Singapore
[6]Division of Neurosurgery, Department of Surgery, Toronto Western Hospital, University of Toronto, Toronto, Ontario, Canada
[7]Institute of Biomedical Engineering, University of Toronto, Toronto, Ontario, Canada
[*]Corresponding authors: xinmu@mpi-halle.mpg.de, wesley.sacher@mpi-halle.mpg.de

February 2, 2026

Supplementary Materials

Microelectrode characterization and analysis

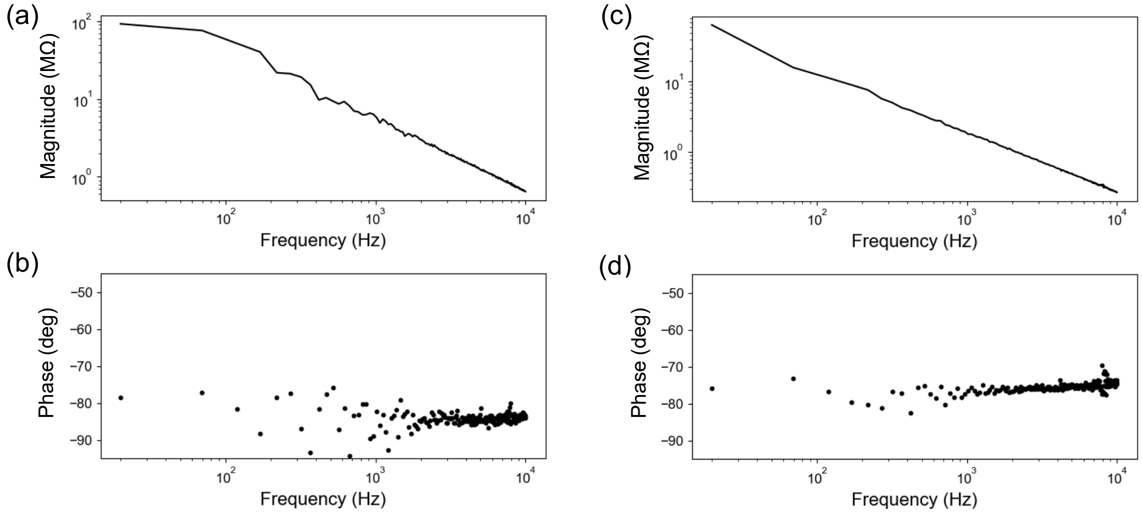

Fig. S1: Electrochemical impedance spectra of a representative microelectrode: (a) magnitude and (b) phase of the impedance before femtosecond-laser surface treatment, and (c) magnitude and (d) phase after laser treatment.

The electrochemical impedance spectra of microelectrodes on neural probes prior to packaging were measured with an impedance analyzer (E4990A, Keysight, Santa Rosa, CA, USA). During impedance measurement, the neural probe shank was immersed in a 1× phosphate buffered saline solution, and the bond pads on the probe base were contacted with a tungsten needle probe. The voltage input from the impedance analyzer was set as 10 mV to avoid electrolysis in the aqueous solution, and the frequency swept from 20 Hz to 10 kHz. More measurement details are reported in Ref. 1. To enable more comprehensive assessment of the electrochemical interface properties, the Bode plots of a representative electrode before and after laser treatment are shown in Fig. S1. The magnitude of electrochemical impedance decreases with frequency, Figs. S1a and c. The magnitude of impedance reduced from 5.75 MΩ to 1.80 MΩ at a frequency of 1 kHz after laser treatment. The phase angle fluctuates more at lower frequency, and the fluctuation becomes smaller after laser treatment, Figs. S1b and d. The phase angle varied between -90° and -75° before laser treatment, and between -80° and -70° after post-processing the electrode surface with femtosecond laser pulses, Figs. S1b and d [2]. Overall, the Bode plots indicate a predominantly capacitive electrochemical interface [3, 4].

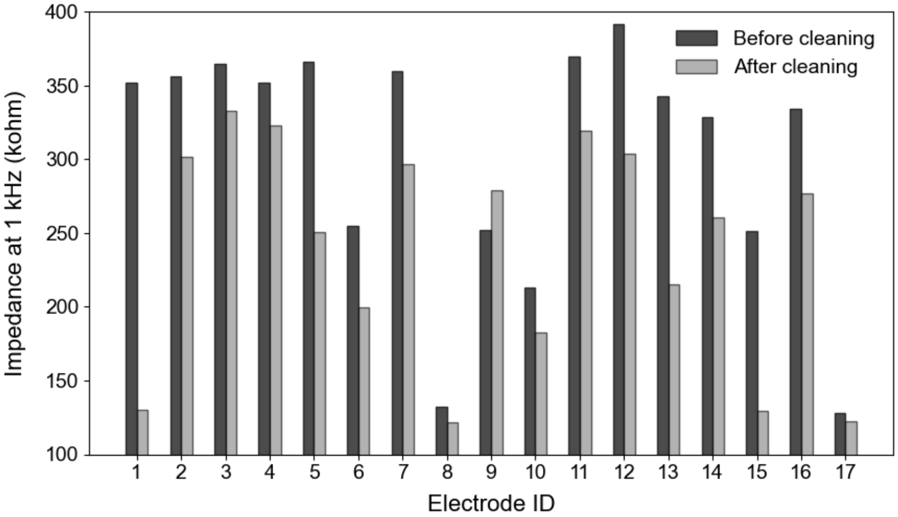

Fig. S2: Comparison of electrode impedance measured before and after neural probe cleaning with a Tergazyme solution following the *in vivo* experiment. One of the 18 electrodes on the neural probe was found to be non-functional during the experiment.

To further investigate the impact of neural probe cleaning with the Tergazyme solution on electrode impedance, we compared the impedance of electrodes from 3 neural probe samples before and after cleaning following the *in vivo* experiments. Figure S2 shows the electrode impedance at 1 kHz from one representative neural probe sample; for 16 of the 17 electrodes (excluding electrode 9), the impedance decreased after immersion in the Tergazyme solution. A similar reduction in electrode impedance after cleaning was observed in 17 of 18 and 15 of 17 electrodes for the other two neural probe samples, respectively. The decreased impedance may result from the restorative effect of Tergazyme cleaning in mitigating biofouling.

To assess signal quality limitations during electrophysiological recording, the crosstalk between microelectrodes on the neural probe shank is estimated. Based on the metal routing trace dimensions described in section "Neural probe design, fabrication, and characterization", the traces are approximated as infinite parallel conductors [5]. Following Ref. 2, the coupling capacitance between adjacent traces per unit length is given by Equation 1:

$$C_c = \epsilon \frac{hL}{d}, \tag{1}$$

where ϵ is the permittivity of the oxide layer, h and L are the trace thickness and length, respectively, and d is the spacing between adjacent traces. The shunting capacitance to ground can be approximated by Equation 2:

$$C_s = \epsilon \frac{wL}{t_b}, \tag{2}$$

where w is the width of the metal trace and t_b is the thickness of bottom oxide layer. The crosstalk

between two recording electrodes with adjacent routing traces can be further defined as [2]:

$$X_e = -20 * \log_{10}(2 + \frac{|Z_c|}{|Z_e|} + \frac{|Z_c|}{\frac{|Z_s|*|Z_L|}{|Z_s|+|Z_L|}}), \tag{3}$$

where $|Zc|$ is the magnitude of the impedance associated with coupling capacitance Cc, $|Ze|$ is the magnitude of the electrode's impedance, $|Zs|$ is the magnitude of the impedance corresponding to the shunting capacitance Cs, and $|Z_L|$ is the magnitude of impedance at the amplifier input capacitance (12 pF for the headstage used in this work [6]). Assuming a 5.23-MΩ electrode impedance at 1 kHz frequency (the mean value for as-fabricated electrodes), the crosstalk between recording electrodes with adjacent traces in either M1 or M2 is -52 dB (the M3 layer was not used for on-shank routing to electrodes). The crosstalk between recording electrodes further reduces to -66 dB and -75 dB for electrodes with impedance of 0.79 MΩ (mean value after laser treatment) and 0.26 MΩ (mean value after *in vivo* experiments). Crosstalk between M1 and M2 traces is similarly low, with the interlaced routing configuration (M2 traces positioned in the gaps between the underlying M1 traces) mitigating the effect of the small inter-layer spacing. Overall, this analysis indicates negligible signal crosstalk between the electrodes during electrophysiological recording.

Neural probe with two microfluidic channels

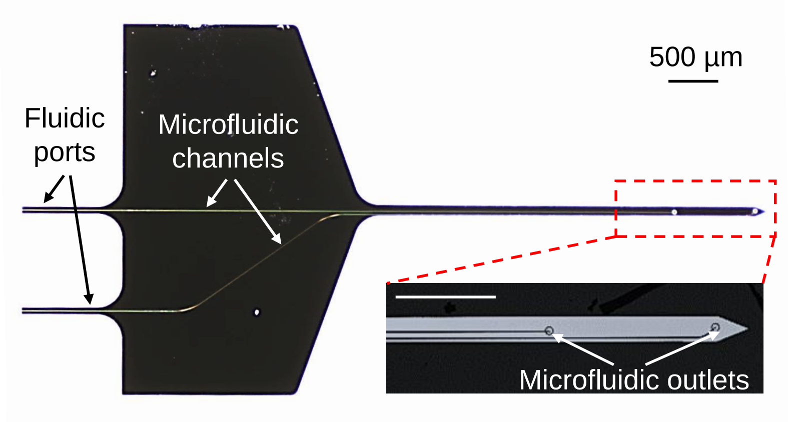

Fig. S3: Micrograph of a fabricated prototype neural probe with two input ports, two embedded microfluidic channels, and two outlets. Scale bars: 500 µm. Optical emitters and electrodes were not patterned on the probe. Two-channel neural probes can be used for delivery of multiple pharmacological agents or for combined microfluidic delivery and sampling.

Multifunctional nanophotonic neural probe system

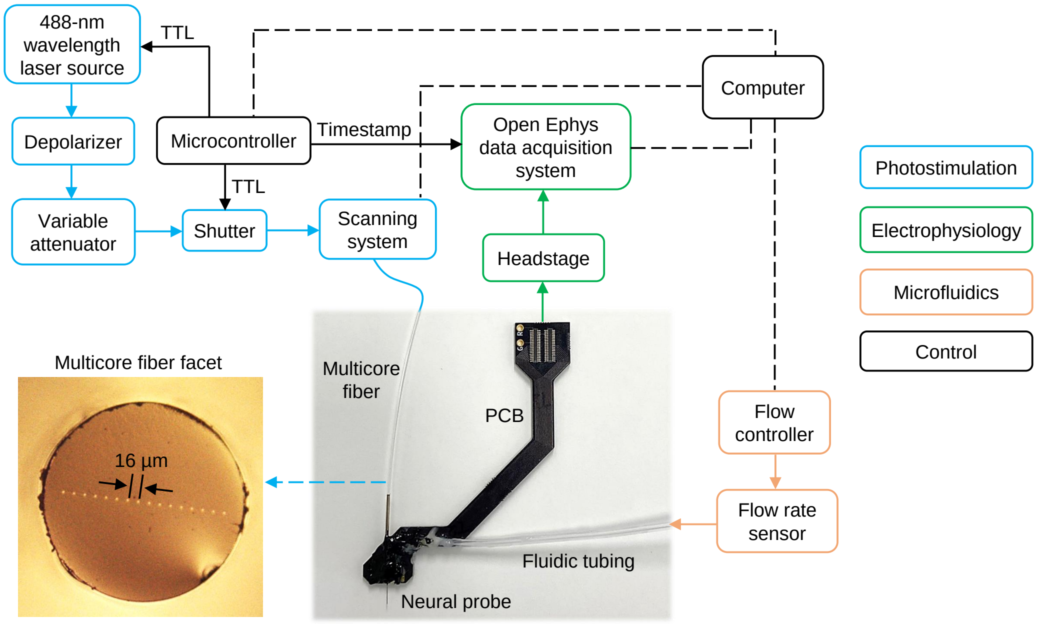

Fig. S4: Schematic of the neural probe system. Laser light is transmitted from a 488-nm wavelength laser source to the neural probe via a depolarizer, variable optical attenuator, optical shutter, optical scanning system, and multicore fiber. The 16-core fiber is aligned and attached to the array of on-chip edge couplers of the neural probe. The scanning system enables selection of the fiber core (and optical emitter) to which light is coupled. The electrophysiological signals recorded by microelectrodes are acquired by an Open Ephys data acquisition system via a heastage and an electrical cable. A microcontroller controls the laser source and the optical shutter with transistor-transistor-logic (TTL) signals, defining the pulse trains for photostimulation. The TTL signals are also transmitted to the Open Ephys system to log the photostimulation timestamps. Fluidic delivery is actuated by a flow controller, with the in-line flow rate sensor capturing flow rates in real time. A laboratory computer provides overall system control through signals sent to the microcontroller, scanning system, and flow controller, while also retrieving recording and timing data from the Open Ephys system. Bottom-left: micrograph of multicore fiber facet. Bottom-middle: photograph of a packaged neural probe.

Control experiments

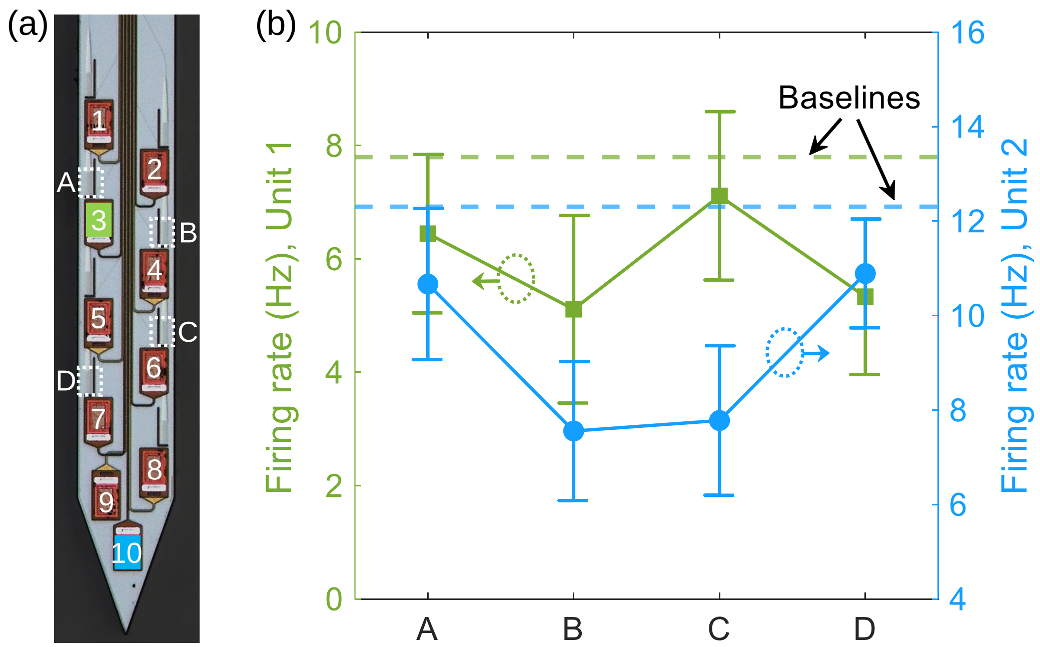

Fig. S5: Photostimulation test in a wild-type mouse. (a) Micrograph of the neural probe shank with selected electrodes and emitters labeled. (b) Mean firing rates of 2 sorted single units (corresponding to electrodes 3 and 10) with photostimulation from 4 emitters (A - D). Mean baseline firing rates were averaged over 10 s before each stimulus. Error bars: standard error of the mean (SEM).

To verify the origin of the evoked spiking response with photostimulation from the neural probe in section "Optogenetic stimulation and electrophysiological recording" (with blue-light-sensitive optogenetic mice), additional photostimulation tests were performed in two wild-type mice (2 to 4 months old). The implant location and photostimulation pattern were identical to section "Optogenetic stimulation and electrophysiological recording", with the exception that a different set of four emitters were selected for sequential addressing (as shown in Fig. S5a). Also similar to section "Optogenetic stimulation and electrophysiological recording", the emission powers ranged from ≈1.0 - 2.4 μW across the four emitters.

Figure S5b shows representative results from one of the control experiments, and similar results were observed in the other control experiment. Mean firing rates of two sorted single units (Unit 1 and Unit 2) with photostimulation from the four emitters are shown. Units 1 and 2 were detected on electrodes 3 and 10, respectively. Compared with the baseline firing rate, increases in firing rates during photostimulation were not observed, indicating the photostimulation effects reported in section "Optogenetic stimulation and electrophysiological recording" were of optogenetic origin rather than a result of tissue heating with illumination. Five additional units were sorted in the experiment, and no significant changes in firing rates with photostimulation were observed.

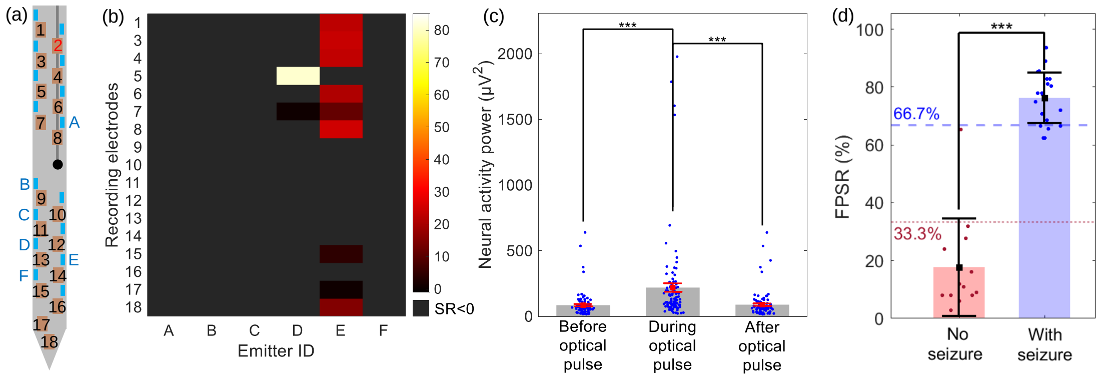

Fig. S6: Control photostimulation tests prior to microfluidic 4-AP injection. (a) Schematic of the probe shank with electrodes and selected emitters labeled. (b) Heatmap of suppression ratios (SRs) on 17 electrode channels with CW optical pulses from 6 emitters. (c) Neural activity power calculated from LFP signals before, during, and after the optical pulses (n=102). (d) Fraction of positive suppression ratios ($FPSR$) in each photostimulation pattern in control tests (before 4-AP injections, n=13, 4 mice) and in seizure suppression tests (after 4-AP seizure induction, n=21, 5 mice, repeated from Fig. 6e). The error bars denote standard deviations in panels (c) and (d). One-tailed non-parametric Mann–Whitney test was used in panels (c) and (d). *** denotes statistical significance with p<0.001.

In four of the five *in vivo* experiments reported in section "Local suppression of 4-AP-induced seizure with continuous-wave photostimulation", the photostimulation pattern was applied repeatedly before the first 4-AP injection. These control tests aimed to confirm the effects of photostimulation prior to the induction of seizure activity. Figure S6 shows the results from one of the four experiments (performed as part of the overall experiment in Fig. 6). The six selected emitters are shown in Fig. S6a and are identical to Fig. 6; the same emission powers were used. The heatmap of suppression ratios (SR, defined in section "Local suppression of 4-AP-induced seizure with continuous-wave photostimulation") during the photostimulation pattern is shown in Fig. S6b. 91 of the data points (emitter-electrode combinations) exhibited $SR < 0$, indicating the photostimulation generally excited neural activity prior to the injections of 4-AP. Figure S6c compares the neural activity power before, during, and after the optical pulses. The neural activity power during photostimulation was significantly higher than pre- and post- stimulation, agreeing with the results of section "Optogenetic stimulation and electrophysiological recording" and the expected photostimulation mechanism (excitation of Channelrhodopsin-2-positive pyramidal cells). As in Fig. 6d, neural activity power pre- and post-stimulation were similar. Also, due to the absence of seizure activity, the amplitude of the neural activity power was lower in these control tests compared to Fig. 6d.

Figure S6d summarizes the fraction of positive suppression ratios ($FPSR$, defined in section "Local suppression of 4-AP-induced seizure with continuous-wave photostimulation") within each photostimulation pattern (trial). The set of control tests (13 trials with 4 mice) is compared to the set of seizure suppression tests in section "Local suppression of 4-AP-induced seizure with continuous-wave photostimulation" (21 trials with 5 mice). During seizure suppression tests (following injections of 4-AP), 18 out of 21 trials had $FPSR \geq 66.7\%$. By contrast, in the control tests, 12 out of

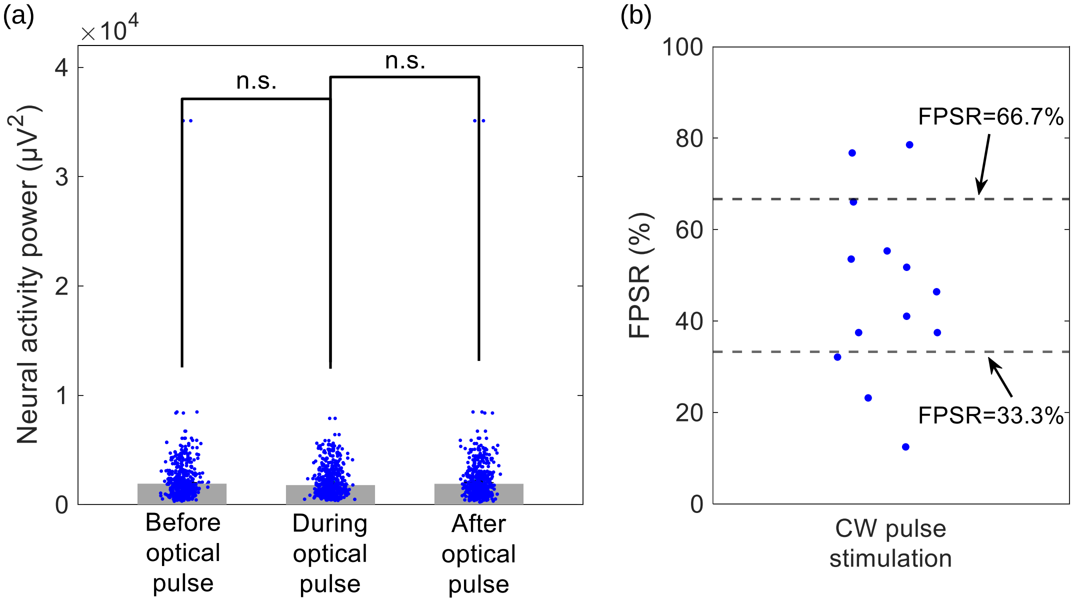

Fig. S7: Optical stimulation alone does not alter neural activity in wild-type mice. (a) Neural activity power before, during, and after the 10-s continuous-wave optical pulses (n=392) in one mouse. The neural activity power was recorded from 14 electrodes during 7 photostimulation trials; light was applied sequentially from 4 emitters during each trial. Two-tailed non-parametric Mann–Whitney test was applied with n.s. denoting no statistical significance with p>0.05. (b) $FPSRs$ calculated across 13 photostimulation trials in 2 wild-type mice. The error bars denote SEM.

13 trials exhibited $FPSR < 33.3\%$, further indicating a general increase in neural activity with photostimulation prior to 4-AP injections.

Control experiments corresponding to the seizure suppression tests in section "Local suppression of 4-AP-induced seizure with continuous-wave photostimulation" were performed on two wild-type mice (2 - 4 months old). The neural probe implant location, composition of the injected solution, and photostimulation pattern (10-s CW pulses emitted in sequence from each of the selected emitters) followed section "Local suppression of 4-AP-induced seizure with continuous-wave photostimulation". Relative to section "Local suppression of 4-AP-induced seizure with continuous-wave photostimulation", a different neural probe was used for these tests, and the set of selected emitters differed in location and number. Emission powers ranged from ≈1.0 - 4.2 μW. The neural probe captured LFP signals during seizure activity induced by the microfluidic injections of 4-AP, and Fig. S7 summarizes the recordings before, during, and after each photostimulation pulse. Seven repetitions (trials) of the photostimulation pattern were performed in the first wild-type mouse, and six were performed in the second mouse. At the implant depth of these experiments, 14 electrodes on the neural probe were within the brain. In Fig. S7a, no statistically significant difference was observed in the neural activity power before, during, and after the photostimulation pulses. Figure S7b shows the calculated $FPSRs$ (defined in section "Local suppression of 4-AP-induced seizure with continuous-wave photostimulation") across all photostimulation trials in both wild-type mice. 61.5% of the trials resulted in $33.3\% < FPSR < 66.7\%$, indicating neither clear

suppression nor promotion effect of seizure activity. Moreover, with $FPSR \geq 66.7\%$ corresponding to clear suppression of seizure activity, only 15.4% of the control trials fell in this range, in contrast to 85.7% with the blue-light sensitive optogenetic mice in section "Local suppression of 4-AP-induced seizure with continuous-wave photostimulation" — indicating that the local suppression of 4-AP induced seizure with CW optical pulses in section "Local suppression of 4-AP-induced seizure with continuous-wave photostimulation" resulted from an optogenetic response instead of tissue heating with photostimulation.

Tests of low- and high-frequency photostimulation patterns for local seizure modulation

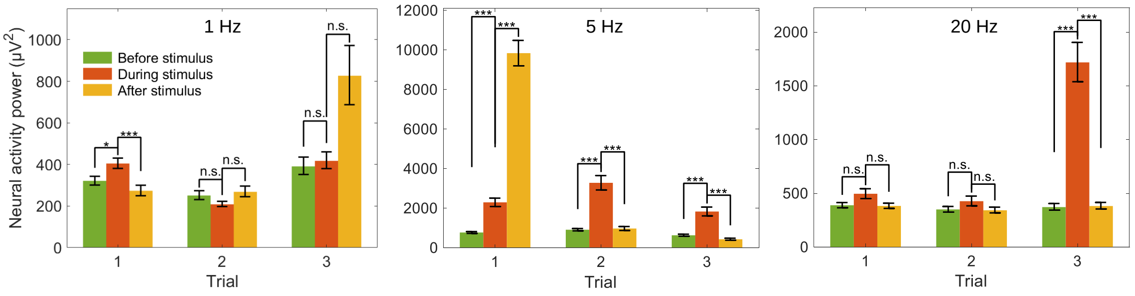

Fig. S8: Neural activity power before, during and after photostimulation pulses at 1 Hz (left), 5 Hz (middle), and 20 Hz (right). Each photostimulation pattern was tested 3 times for each selected emitters during the experiment (trials 1 - 3). The error bars denote SEM. Two-tailed non-parametric Mann–Whitney test was applied with *** denoting statistical significance with $p<0.001$ and * for $p<0.05$, n.s. stands for no statistical significance with $p\geq0.05$.

In addition to the CW photostimulation pattern tested in section "Local suppression of 4-AP-induced seizure with continuous-wave photostimulation", additional photostimulation patterns from neural probes were tested for seizure suppression following microfluidic injections of 4-AP (with the same procedure of section "Local suppression of 4-AP-induced seizure with continuous-wave photostimulation"). These tests spanned low- (1 Hz and 5 Hz) and high- (20 Hz) frequency photostimulation pulse trains. The parameters of the three additional photostimulation patterns are summarized in Table S1; three different probes were used across the tests, and the number of selected emitters also varied. Three repeats of the pattern were performed for each selected emitter (trials 1 - 3). Figure S8 compares the neural activity power before, during, and after each stimulation pattern (calculated across all electrodes, as in section "Local suppression of 4-AP-induced seizure with continuous-wave photostimulation"). All tests were performed during seizure activity. Overall, no reduction in neural activity power during photostimulation (i.e. seizure suppression) was observed across the three additional photostimulation patterns.

Table S1: Summary of low- and high-frequency photostimulation patterns (adapted from the references listed).

**Frequency (Hz)**	**Pulse width (ms)**	**Recovery time (s)**	**Number of pulses**	**Number of emitters**	**Emission power range (μW)**	**Ref.**
1	5	120	120	4	≈4.3 - 5.3	[7]
5	50	120	600	1	≈3	[8]
20	5	5	200	1	≈7.8	[9]

References

1. Chen, F.-D. *et al.* Implantable nanophotonic neural probes for integrated patterned photostimulation and electrophysiological recording. *npj Biosensing* **2,** 15 (2025).
2. Rios, G. Nanofabricated neural probe system for dense 3-D recordings of brain activity. *PhD thesis* (2016).
3. Orazem, M. E., Pébère, N. & Tribollet, B. Enhanced graphical representation of electrochemical impedance data. *J. The Electrochem. Soc.* **153,** B129 (2006).
4. Huang, J., Li, Z., Liaw, B. Y. & Zhang, J. Graphical analysis of electrochemical impedance spectroscopy data in Bode and Nyquist representations. *J. Power Sources* **309,** 82–98 (2016).
5. Sellberg, F. Simple determination of all capacitances for a set of parallel microstrip lines. *IEEE Trans. Microw. Theory Tech.* **46,** 195–198 (2002).
6. Nelson, M. J., Valtcheva, S. & Venance, L. Magnitude and behavior of cross-talk effects in multichannel electrophysiology experiments. *J. Neurophysiol.* **118,** 574–594 (2017).
7. Ladas, T. P., Chiang, C.-C., Gonzalez-Reyes, L. E., Nowak, T. & Durand, D. M. Seizure reduction through interneuron-mediated entrainment using low frequency optical stimulation. *Exp. Neurol.* **269,** 120–132 (2015).
8. Soper, C., Wicker, E., Kulick, C. V., N'Gouemo, P. & Forcelli, P. A. Optogenetic activation of superior colliculus neurons suppresses seizures originating in diverse brain networks. *Neurobiol. Disease* **87,** 102–115 (2016).
9. Chiang, C.-C., Ladas, T. P., Gonzalez-Reyes, L. E. & Durand, D. M. Seizure suppression by high frequency optogenetic stimulation using *in vitro* and *in vivo* animal models of epilepsy. *Brain Stimul.* **7,** 890–899 (2014).
